# Protective effects of *Erythronium japonicum* and *Corylopsis coreana* Uyeki extracts against 1,3-dichloro-2-propanol-induced hepatotoxicity in rats

**DOI:** 10.1186/s42649-020-00049-0

**Published:** 2020-12-02

**Authors:** Seunghyun Kim, Hee-Ock Boo, Taeho Ahn, Chun-Sik Bae

**Affiliations:** 1 College of Veterinary Medicine, Chonnam National University, Gwangju, 61186 South Korea; 2 Wellphyto Co. Ltd, Gwangju, 61950 South Korea

**Keywords:** *Erythronium japonicum*, *Corylopsis coreana* Uyeki, 1,3-dichloro-2-propanol, Liver, Rats

## Abstract

*Erythronium japonicum* (*E. japonicum*) and *Corylopsis coreana* Uyeki (*C. coreana* Uyeki, Korean winter hazel) have been shown to significantly decrease 1,3-dichloro-2-propanol (1,3-DCP)-induced generation of reactive oxygen species and CYP2E1 activity in HuH7, human hepatocytes. In this study, we expanded upon the previous study and investigated the effects of *E. japonicum and C. coreana* Uyeki extracts on 1,3-DCP-induced liver damage in rats. The pre-treatment of rats with these extracts alleviated a decrease in body weight and reduced 1,3-DCP-induced increase in catalytic activities of hepatic enzymes, such as aspartate aminotransferase and alanine aminotransferase, in the serum. Moreover, treatment with the extracts restored the 1,3-DCP-induced decreases in anti-oxidant enzyme activities, such as the activities of superoxide dismutase and catalase, in the rat liver. Histopathological studies also strongly supported the results of enzyme activities. These results suggest a possibility that the extracts of *E. japonicum and C. coreana* Uyeki can be a remedy for alleviating 1,3-DCP-induced liver damage in animals.

## Introduction

1,3-Dichloro-2-propanol (1,3-DCP) is a high-production-volume chemical that was shown to have toxicological properties by the National Institute of Environmental Health Sciences. There is concern regarding the exposure of humans in the workplace during the manufacturing and use of chemicals such as epichlorohydrin, 1,2,3-trichloropropane, and 1,3-dichloropropene (Hammond and Fry 1999). In particular, exposure to 1,3-DCP may occur through the diet via the addition of hydrochloric acid-hydrolyzed vegetable protein, and through drinking water with added epichlorohydrin polyamine polyelectrolytes used as flocculants and coagulants in water purification (Nyman et al. 2003). In a previous study in rats, the intraperitoneal (i.p.) injection of 1,3-DCP induced drowsiness, liver injury, significant increase in the activity of serum alanine aminotransferase (ALT), decreased white blood cell and platelet counts, and increased blood clotting time (Katoh et al. 1998).

*Erythronium japonicum* is a perennial herb belonging to the fawn lily family, and is found throughout Japan, Korea, northeast China, Sakhalin, and the Kurile Islands. Regarding its pharmacological properties, Heo et al. (2007) reported that *E. japonicum* extract has 2,2-diphenyl-1-picrylhydrazyl free radical scavenging activity and exerts anti-proliferative effects in human colorectal carcinoma cells. *Corylopsis coreana* Uyeki (*C. coreana* Uyeki, Korean winter hazel), belonging to the Hamamelidaceae family, is cultivated as an ornamental plant and is native to South Korea. Some species of the genus *Corylopsis*, such as *Hamamelis virginiana* (witch hazel), have been used as traditional herbal medicines for the treatment of irritated skin and inflammatory disease (Kim et al. 2013). In addition, the plant exhibits anti-irritant, anti-inflammatory, and anti-tumor effects (Korting et al. 1993; Deters et al. 2001; Lizárraga et al. 2008).

In a recent study using the extracts of *E. japonicum* and *C. coreana* Uyeki, 1,3-DCP-induced generation of reactive oxygen species and CYP2E1 activity was shown to significantly decrease in HuH7, human hepatocytes (Bae et al. 2018). Boo et al. (2013) also suggested that the extracts exhibited strong inhibitory activities against α-amylase and α-glucosidase and scavenged 1,1-diphenyl-2-picrylhydrazyl radicals in vitro.

In the present study, we evaluated the effects of *E. japonicum and C. coreana* Uyeki extracts against liver damage induced by 1,3-DCP treatment in rats.

## Materials and methods

### Animals and environmental conditions

Seven-week-old male Sprague–Dawley rats, weighing 269.5 ± 16.54 g, were provided by Samtako Bio Korea Co., Ltd. (Osan, South Korea) and were used after 1 week of quarantine and acclimatization. They were housed in an air-conditioned room with a 12 h light/dark cycle under controlled illumination (200–300 lx), temperature (23 ± 2 °C), and humidity (55 ± 10%). Tap water and commercial rodent diet (Samyang Feed, Wonju, South Korea) were provided ad libitum. The Institutional Animal Care and Use Committee of Chonnam National University approved the protocols for the animal study, and the animals were cared for in accordance with the Guidelines for Animal Experiments of Chonnam National University.

### Test chemicals and treatment

*E. japonicum* and *C. coreana* Uyeki plant extracts were provided by WellPhyto Co. (Gwangju, South Korea). 1,3-DCP was purchased from Sigma Aldrich (Merck Millipore, Darmstadt, Germany). The extracts (2 or 5%) were administered orally in 10 mL/kg doses, calculated based on the average daily intake of the animals, at a regular time once per day for 7 days. To induce liver injury following the final dose of the extract, rats were administered 1,3-DCP aqueous solution (1 mL/kg) at doses of 80 mg/kg as a single i.p. injection.

### Experimental groups

The animals were randomly divided into six experimental groups (*n* = 7 per group) as follows: 1) negative control group; 2) 1,3-DCP-treated group that received a single dose of 1,3-DCP, which has been shown to induce acute hepatotoxicity in rats (Katoh et al. 1998); 3) 2% *E. japonicum* extract group; 4) 5% *E. japonicum* extract group; 5) 2% *C. coreana* Uyeki extract group; and 6) 5% *C. coreana* Uyeki extract group. Groups 3–6 were treated with the plant extract orally once daily during the experimental period.

### Clinical observation and body weight

All animals were observed once daily for mortality and clinical signs of reaction to the treatment. The body weight and weight gain of each animal were measured at the end of treatment and before the animal was sacrificed.

### Serum biochemical analysis

The animals were anesthetized with a combination of xylazine hydrochloride (Rompun; Bayer Korea, Korea; 10 mg/kg) and ketamine (ketamine HCl; Yuhan Co., South Korea; 40 mg/kg) after the 1,3-DCP injection. Blood samples were collected by venipuncture from the posterior vena cava. Each sample was centrifuged at 3000 rpm for 15 min within 30 min after collection, after which the top serum layer was removed. Aspartate aminotransferase (AST) and alanine aminotransferase (ALT) in the serum were measured using an auto-analyzer (Dri-chem 4000i; Fujifilm Co., Tokyo, Japan).

### Superoxide dismutase (SOD) and catalase (CAT) assays

After the collection of blood samples, all animals were sacrificed by blood-letting. The liver was harvested and the absolute and relative (organ-to-body) weights were measured. Samples from the liver tissue were washed in phosphate buffered saline and stored at − 80 °C for the measurement of SOD and CAT. The frozen liver samples were homogenized in a glass-Teflon homogenizer with 50 mM phosphate buffer (pH 7.4) to obtain 1:9 (w/v) whole homogenate. The homogenates were centrifuged at 11,000 ×*g* for 15 min at 4 °C to remove any cell debris. SOD activity was determined according to the method of Beauchamp and Fridovich (1971). The final supernatant, containing 50 mM carbonic buffer (pH 10.2), 0.1 mM Na_2_-EDTA, 0.1 mM xanthine, and 0.025 mM nitroblue tetrazolium (NBT), was illuminated at 25 °C for 10 min. Then, xanthine oxidase (3.3 × 10^− 6^ mM) was added to the reaction mixture, and the reduction of NBT was measured spectrophotometrically at 560 nm. CAT activity was determined using the method of Aebi (1984). A decrease in the absorbance of H_2_O_2_ was measured spectrophotometrically at 240 nm in 1 mL of reaction mixture containing 10 mM H_2_O_2_ and 20 μL supernatant in 50 mM potassium phosphate buffer (pH 7.0). CAT activity was expressed as l mole of H_2_O_2_ decomposed mg protein^− 1^ min^− 1^ at pH 7.0 at 25 °C. Total protein concentrations were determined using Bradford assay with bovine serum albumin as the protein standard.

### Histological analysis

Dissected liver tissues were fixed in 10% neutral buffered formalin and embedded in paraffin. The paraffin-embedded sections were cut into 4-μm thickness, deparaffinized, and rehydrated using standard techniques. The liver sections were stained with hematoxylin and eosin. Histological changes, such as hepatocyte degeneration/necrosis and inflammatory cell infiltration of the liver, were observed using a microscope.

### Statistical analysis

The data were analyzed by using GraphPad InStat version 3.0 (GraphPad Software, Inc., La Jolla, CA, USA). The results were expressed as the mean ± standard deviation. Differences between groups were analyzed by one-way analysis of variance followed by Dunnett’s multiple comparison test. A *P* value of *<* 0.05 was considered to indicate a statistically significant difference.

## Results and discussion

As a preliminary test prior to the evaluation of the anti-oxidative effects of the *E. japonicum* and *C. coreana* Uyeki extracts, we first examined the clinical signs in rats induced by the extracts, such as effects on mortality, body weight, and the liver. In toxicological studies, body and organ weights generally indicate the presence of potentially toxic chemicals (Kim et al. 2004; Lee et al. 2009). Treatment-related mortality and clinical signs were not observed in all rats tested during the study period (results not shown). In contrast to the gain in body weight observed in rats in the control group, the rats treated with 1,3-DCP alone showed the most significant decrease in body weight (Table 1). However, 2% and 5% *E. japonicum* (or *C. coreana* Uyeki) treatment after 1,3-DCP administration resulted in a slight decrease in the weight, which was not statistically significant. Therefore, these results suggested that *E. japonicum* or *C. coreana* Uyeki extracts may exert protective effects against the decrease in body weight induced by 1,3-DCP. However, a dose-dependent protective effect was not observed, as the extent of weight loss was similar at both treatment concentrations. In contrast to the change in body weight, no significant differences was observed in the relative weights of the rat liver (weight ratio of body to tissue) in all groups tested (results not shown).

**Table 1:** Body weight changes in male rats

Parameters	Group					
	Control	1,3-DCP	2% *Erythronium japonicum*	5% *Erythronium japonicum*	2% *Corylopsis coreana* Uyeki	5% *Corylopsis coreana* Uyeki
Before 1,3-DCP treatment	296.3 ± 12.45	310.0 ± 14.18	302.3 ± 26.79	311.1 ± 7.71	313.9 ± 13.90	311.7 ± 17.19
After 1,3-DCP treatment	303.5 ± 16.41	297.5 ± 12.01	296.2 ± 29.55	304.5 ± 13.35	308.5 ± 10.25	308.9 ± 18.01
Weight gain	8.6 ± 2.48	−12.6 ± 4.87^*,^^**^	− 6.1 ± 7.64^*,**^	− 6.6 ± 8.89^*,**^	− 5.3 ± 5.52^*,**^,^†^	− 2.8 ± 5.83^*,**^,^††^

1,3-DCP, 1,3-dichloro-2-propanol

Values are presented as means ± SD (g) of seven rats

Control represents a group without any treatments

^*^Significant difference at the *P* < 0.05 level compared with the Control group

^**^Significant difference at the *P* < 0.01 level compared with the Control group

^†^Significant difference at the *P* < 0.05 level compared with the 1,3-DCP group

^††^Significant difference at the *P* < 0.01 level compared with the 1,3-DCP group

The results of serum biochemical analyses are summarized in Table 2. The marked increases in serum AST and ALT levels are attributed to severe damage in hepatic tissue membranes, because they are located in the cytoplasm and are released from cells after autolytic breakdown or cellular necrosis (Khan et al. 2012). The rats treated with 1,3-DCP showed a significant increase in the catalytic activities of serum AST and ALT, which indicated the induction of liver damage in the animals. However, the treatment of rats with 2% and 5% *E. japonicum* extract resulted in a significant decrease in the activities of these enzymes. Moreover, treatment with *C. coreana* Uyeki extract caused a more drastic reduction in enzyme activities, in a dose-dependent manner, and the AST and ALT levels almost reached the normal range following treatment with 5% *C. coreana* Uyeki extract. These results clearly suggested that *E. japonicum* and *C. coreana* Uyeki extracts may protect the liver against 1,3-DCP-induced damage in rats. However, the dose-dependency of extract was not observed in the *E. japonicum*-treated group. Therefore, further studies should be performed to elucidate the relationship between the attenuation of liver damage and the extract concentration used for treatment. Moreover, the molecular mechanism of the protective effect of extract remains unclear.

**Table 2:** Effects of test materials on serum aspartate aminotransferase (AST) and alanine aminotransferase (ALT) activities in rats

Parameters	Group					
	Control	1,3-DCP	2% *Erythronium japonicum*	5% *Erythronium japonicum*	2% *Corylopsis coreana* Uyeki	5% *Corylopsis coreana* Uyeki
AST	165.6 ± 27.87	16,326.7 ± 2815.80^*,**^	1816.0 ± 2793.30^††^	2524.0 ± 2308.00^††^	828.0 ± 520.88^††^	360.0 ± 247.79^††^
ALT	65.3 ± 34.89	11,853.3 ± 7052.20^*,**^	1516.0 ± 1805.60^††^	1720.0 ± 2188.01^††^	972.0 ± 695.93^††^	404.0 ± 228.65^††^

1,3-DCP, 1,3-dichloro-2-propanol

Values are presented as means ± SD of seven rats

^*^Significant difference at the *P* < 0.05 level compared with the Control group

^**^Significant difference at the *P* < 0.01 level compared with the Control group

^†^Significant difference at the *P* < 0.05 level compared with the 1,3-DCP group

^††^Significant difference at the *P* < 0.01 level compared with the 1,3-DCP group

SOD and CAT are antioxidant enzymes known to scavenge free radicals in cells, and their catalytic activities indicate the oxidative status such as lipid peroxidation in organs with extensive cellular damage (Jurczuk et al. 2004). SOD and CAT assay with the liver homogenates was performed for all groups tested, and the results are summarized in Table 3. The activities of SOD and CAT were significantly diminished by treatment with 1,3-DCP. Based on this result and the increased values of AST and ALT shown in Table 2, it could be anticipated that 1,3-DCP induces liver toxicity by cellular necrosis, which may be associated with the increased production of reactive oxygen species due to decreased SOD and CAT activities in the liver (Haratake et al. 1993). In contrast, the activity of SOD and CAT were significantly restored by treatment with 5% *E. japonicum* or 5% *C. coreana* Uyeki extracts. However, the recovery effects were not apparent, or were marginal, in groups treated with 2% extracts. Nevertheless, these results indicated that the extracts of *E. japonicum* and *C. coreana* Uyeki play an important role in the recovery and/or protective effect against a decrease in anti-oxidant enzyme activities in the rat liver. The results also suggested that the reduction in AST and ALT activities in the serum following treatment with the extracts may be related with the restored enzymatic levels of SOD and CAT. However, the molecular mechanisms underlying the extract-mediated changes in enzyme activities of SOD and CAT, as well as AST and ALT, could not be elucidated through the current experiments.

**Table 3:** Superoxide dismutase (SOD) and catalase (CAT) activities in the rat liver

Parameter	Group					
	Control	1,3-DCP	2% *Erythronium japonicum*	5% *Erythronium japonicum*	2% *Corylopsis coreana* Uyeki	5% *Corylopsis coreana* Uyeki
SOD (units/mg protein)	174.6 ± 33.88	83.3 ± 14.01^*,**^	93.1 ± 16.17^*,**^	104.4 ± 16.64^*,**^	77.1 ± 18.64^*,**^	108.4 ± 28.77^*,**^
CAT (units/mg protein)	11.49 ± 1.415	2.40 ± 0.626^*,**^	2.92 ± 0.638^*,**^	4.59 ± 0.712^*,**,††^	2.77 ± 0.339^*,**^	5.65 ± 0.775^*,**,††^

1,3-DCP, 1,3-dichloro-2-propanol

Values are presented as means ± SD of seven rats

^*^Significant difference at the *P* < 0.05 level compared with the Control group

^**^Significant difference at the *P* < 0.01 level compared with the Control group

^†^Significant difference at the *P* < 0.05 level compared with the 1,3-DCP group

^††^Significant difference at the *P* < 0.01 level compared with the 1,3-DCP grou

**Fig. 1:**
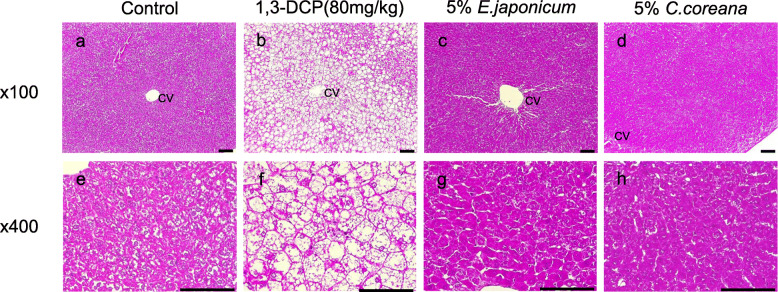
Histopathological features on the protective effects of *Erythronium japonicum* and *Corylopsis coreana* Uyeki against a model of 1,3-dichloro-2-propanol (1,3-DCP)-induced hepatotoxicity. Representative photographs of liver sections of **a**, **e** control showing glycogen vacuolation and **b**, **f** 1,3-DCP (80 mg/kg) showing various histopathological alterations characterized by degeneration/necrosis of hepatocytes around the central vein region, vacuolation, inflammatory cell infiltration, hemorrhage. Rats treated with 1,3-DCP at does of **c**, **g** 5% *E.japonicum,* **d**, **h** 5% *C.coreana.* Hematoxylin and eosin stain; Central vein; scale bar = 50 μm (× 100, × 400); 1,3-DCP: 1,3-dichloro-2-propanol

To obtain further insight into 1,3-DCP-induced liver damage and the possible protective effect of *E. japonicum* and *C. coreana* Uyeki extracts on hepatic injury, we investigated the histopathological changes in the liver sections. It has been known that 1,3-DCP induces histopathological changes in the rat liver, including zonal necrosis of the centrilobular space, destruction of the sinusoidal structure, and increase in eosinophilic cellular debris and inflammatory cell infiltration in the necrotic areas (Choi et al. 2009; Stott et al. 1997). In the present study, the necrosis of hepatocytes and infiltration of inflammatory cells were observed around the central vein region of the rats in 1,3-DCP-treated group (80 mg/kg; Fig. 1b, f) compared with the normal liver tissue of control group (Fig. 1a, e). These are the typical histopathological features of liver injury caused by 1,3-DCP. However, the degree of hepatocyte necrosis and inflammation was alleviated around the central vein region of experimental groups treated with 5% *E. japonicum* (Fig. 1c, g) and 5% *C. coreana* Uyeki extracts (Fig. 1d, h). Based on these results, we concluded that 1,3-DCP-induced hepatic lesions were reduced by treatment with both extracts. The appearance of the hepatocytes in the groups treated with the extracts were similar to that of the control group, which further support our conclusion.

It has been suggested that chlorogenic acid from *E. japonicum* is a candidate compound with protective function against oxidative stress-induced hepatotoxicity (Seo et al. 2016). Four marker compounds from *C. coreana* Uyeki have been identified: bergenin (17.5%, w/w); isosalipurposide (8.6%, w/w); quercitrin (1.6%, w/w); and quercetin (0.05%, w/w). Bergenin has a protective effect against d-galactosamine-induced hepatotoxicity in rats (Lim et al. 2001). Isosalipurposide, a chalcone compound, exerts a cytoprotective effect against oxidative injury through Nrf2 activation (Han et al. 2015). Quercitrin and quercetin protect the liver from acetaminophen-induced injury (Shanmugam et al. 2016; Xie et al. 2016). Quercitrin is a glycoside formed from quercetin, which is known to deplete the heme pool and induce carbon monoxide release, thereby limiting the expression and activity of CYP2E1, a well-known enzyme that induce oxidative stress (Tang et al. 2013; Leung and Nieto 2013). These results, therefore, suggest that certain component(s) in these plant extracts may alleviate the 1,3-DCP-mediated hepatotoxicity of rats.

## Conclusion

*E. japonicum and C. coreana* Uyeki extracts alleviated the 1,3-DCP-induced decrease in body weight and the enzyme activities of AST and ALT in the rat serum. In addition, the extracts restored the decreased activities of SOD and CAT in the rat liver homogenates. These results indicated the protective effect of the extracts on 1,3-DCP-mediated liver injury and this conclusion was further supported by histopathological results. Collectively, the present study provide a possibility that the extracts of *E. japonicum* and *C. coreana* Uyeki may be applied to remedy of 1,3-DCP-induced hepatotoxicity in animals.

## Data Availability

Materials described in the manuscript, including all relevant raw data, will be freely available to any scientist wishing to use them for non-commercial purpose.

## Abbreviations

*E. japonicum*: Erythronium japonicum
*C. coreana* Uyeki: Corylopsis coreana Uyeki
1,3-DCP: 1,3-dichloro-2-propanol
AST: Aspartate aminotransferase
ALT: Alanine aminotransferase
SOD: Superoxide dismutase
CAT: Catalase
